# Putative Role of AMPK in Fetal Adaptive Brain Shut-Down: Linking Metabolism and Inflammation in the Brain

**DOI:** 10.3389/fneur.2014.00150

**Published:** 2014-08-11

**Authors:** Martin G. Frasch

**Affiliations:** ^1^Department of Obstetrics and Gynaecology, CHU Ste-Justine Research Center, Université de Montréal, Montreal, QC, Canada; ^2^Department of Neurosciences, CHU Ste-Justine Research Center, Université de Montréal, Montreal, QC, Canada

**Keywords:** fetus, neuroinflammation, EEG, microglia, neurons, metabolism, sheep

In a companion article of the same Research Topic, we present findings on the relationship of fetal adaptive brain shut-down and neuroinflammation during hypoxic acidemia (1). The findings are derived from the chronically instrumented non-anesthetized near-term ovine fetus model with and without chronic hypoxia (defined as arterial O_2_Sat < 55%) subjected to umbilical cord occlusions (UCOs) of increasing severity. This model mimics human labor and is useful for studying the process of worsening acidemia that may precipitate perinatal brain injury. While the neuroinflammation overall decreases between 24 and 48 h post UCOs, the relationship between the degree of neuroinflammation and the timing of the adaptive brain shut-down reverses between these two time points, raising the question as to the underlying physiology.

We propose that adaptive brain shut-down in the fetus, evidenced by changes in EEG, may be mediated via adenosine monophosphate kinase (AMPK) signaling due to its controlling influence over cellular metabolism and interaction with inflammatory signaling pathways. By way of background, consider that the intracellular energy-sensor AMPK plays a key role in cellular metabolism, increases in cellular AMP/ATP ratio result in activation of AMPK via its phosphorylation (2, 3). pAMPK reduces ATP-consuming processes and promotes ATP-producing processes. Consequently, neuronal pAMPK decreases during the relatively less energy-consuming NREM sleep state, which is associated with increased EEG delta wave activity and ATP increase in adult rats (4). Fetal adaptive neuronal shut-down with worsening acidemia may also be mediated via adenosine A1 receptors (1, 5, 6). Notably, a combination of both A1 and AMPK signaling is also a plausible mechanism leading to adaptive brain shut-down. First, Gadalla et al. observed that 5-aminoimidazole-4-carboxamide riboside (AICA riboside), a compound with neuroprotective properties thanks to the AMPK activation, has an additional neuroprotective effect under metabolic stress via competition with adenosine for uptake by the nucleoside transporter leading to an increase of extracellular adenosine and subsequent activation of A1 receptors (7). Second, endogenous extracellular adenosine in physiological concentrations is, in turn, equally able to activate AMPK, an effect requiring active nucleoside transporters, such as CNT2 (8, 9). Both AMPK and A1 receptor activation result in suppression of the more energy-consuming glutamatergic excitatory synaptic neurotransmission (i.e., as opposed to GABAergic inhibitory signaling contributing only ~20% to the neuronal oxidative energy metabolism) (10, 11). Either way, the result would be a relative increase of intracellular ATP and decreasing AMPK levels.

Sag et al. demonstrated *in vitro* that AMPK signaling and pro-inflammatory mediators in macrophages are mutually coupled via negative feedback. AMPK suppresses pro-inflammatory responses such as lipopolysaccharide (LPS)-induced production of TNF-α and IL-6 and promotes macrophage polarization to an anti-inflammatory functional phenotype with increased production of IL-10 (12). Exposure of macrophages to pro-inflammatory cytokines increases AMPK dephosphorylation, while exposure to anti-inflammatory cytokines results in rapid AMPK phosphorylation, i.e., activation (12). Activation of toll-like receptor (TLR) 4 on macrophages by LPS and resultant NF-κB pathway activation lead to a loss of AMPK phosphorylation (13). Hence, the effects of AMPK on the regulation of inflammatory status indicate that the presence of AMPK and its activation are important to counteract inflammation. Similarly, *in vivo* AMPK is down-regulated in all immune cells during experimental autoimmune encephalomyelitis (EAE), the animal model of the autoimmune disease multiple sclerosis (14). Neuronal AMPK is widely expressed in the embryonic and adult rat brains *in situ* and promotes neuronal survival under conditions of hypoglycemia *in vitro* (15).

Adenosine monophosphate kinase activity and its anti-inflammatory consequences have been studied in the context of chronic hypoxia. Chronic hypoxia up-regulates pAMPK *in vitro* in healthy neonatal rat neuronal slice cultures, in the human glioblastoma cells and *in vivo* in the adult rats’ pulmonary vasculature (7, 16, 17). Lactate is a principal energy source for neurons, especially in the developing brain (11, 18, 19). However, excess lactate within the extracellular space of the brain contributes to neuronal injury (3, 19). Recently, AMPK was also shown to play an important role in controlling the degree of cellular inflammation in various cell types including glial cells, thus linking cellular metabolism and inflammation (2, 3, 20). Brain regional lactate acidosis increases neuronal intracellular pAMPK levels (21). At the same time, pAMPK also restricts microglial activation via the IFN-γ signaling pathway decreasing expression of STAT1-inducible inflammatory cytokines in adult mice (2) and, anti-inflammatory effects of AMPK activation have been demonstrated on NF-κB pathway in the primary glial cultures, notably from 1 to 3 days old rat pups, and *in vivo* in adult rats (22).

In seeming contrast to the above cited work, acute brain ischemia in adult male gerbils results in regional (CA1) transient pAMPK and lactate increases, ATP depletion, neuronal death, and microglial *activation* [as opposed to *suppression* of microglial secretory cytokine activity shown by Meares et al. and Giri et al. (3)]. These traits are reversed if an AMPK inhibitor is administered (3). Notably, these authors provided indirect evidence that cortical neuronal pAMPK increases within 90 min post insult, probably to compensate for lack of ATP, while the glial AMPK induction follows within 5 days, when neuronal death is observed. The seemingly contradictory findings regarding the effect of AMPK on neuroinflammation may result from the different animal models used (septic versus aseptic neuroinflammation), varying temporal profiles (acute versus chronic), and neuroinflammatory phenotyping (cytokine secretion versus cell morphology).

In light of the discussed AMPK physiology, it is intriguing to speculate that at 24 h post UCOs, AMPK-mediated neuronal shut-down correlates to decreased brain regional lactate levels, leading to a pronounced decrease in neuroinflammation. In contrast, at 48 h post UCOs, the relationship between the degree of neuroinflammation and the timing of the adaptive brain shut-down may be reversed due to several reasons. First, the fetal brain may be less capable of metabolizing lactate under conditions of pre-existing hypoxia with reduced metabolism and ATP reserves unable to sustain pAMPK activation beyond an acute response. Second, at 48 h post UCOs, regional lactate accumulation may have occurred, in addition to AMPK and inflammatory mediators. Third, one of the side effects of AMPK activation may be an increase in lactate production due to glycolysis, which may contribute to tissue injury (3). While future studies will have to validate these mechanisms in the perinatal brain, it seems plausible that lactic acidosis has the potential to induce variable degrees of microglial activation and neuronal shut-down in an AMPK-dependent manner, in chronically hypoxic fetuses with worsening acidemia. Further investigations are needed into the potential of intrapartum EEG–FHR monitoring to aid detection of adaptive brain shut-down to improve early postnatal diagnostic and therapeutic strategies, such as selecting at-risk newborns for hypothermic interventions to decrease cerebral metabolism (6, 23).

## Conflict of Interest Statement

Martin G. Frasch is an inventor of the related patent application entitled “EEG Monitor of Fetal Health” including U.S. Patent Application Serial No. 12/532,874 and CA 2681926 National Stage Entries of PCT/CA08/00580 filed March 28, 2008, with priority to US provisional patent application 60/908,587, filed March 28, 2007. No other disclosures have been made.
